# Supplementary material for: Content-rich biological network constructed by mining PubMed abstracts
Source: BMC Bioinformatics. 2004 Oct 8;5:147. doi: 10.1186/1471-2105-5-147 (PMC528731; doi:10.1186/1471-2105-5-147)
Supplement: Additional File 5 — The original Chilibot query results of the term "long-term potentiation (LTP)" and 22 other terms, limiting the latest references analyzed to the years 1990, 1995, 2000, and 2004. [file 1471-2105-5-147-S5.bz2 › chilibotAdditionalFile5/ltp2004/html/NMDA_AMPA.html]

 


 **NMDA** and **AMPA** 
  
Found 3211 abstracts in PubMed,  **30 abstracts were retrieved and analyzed**.  


---

 Search Google  |
 PDF files only 
|  EDU domain only 

---

**Interactive relationship** (e.g. stimulation, inhibition, etc)

- Propofol produced antinociception through an interaction with spinal  **NMDA**  and  **AMPA**  receptors in mice.  Ref: 14704116 Acta Pharmacol Sin, 2004
- In the long term, when the map had degraded, responses were readily evoked and predominantly  **AMPA**  kainate receptor mediated although some  **NMDA**  mediated activity and NR1 expression remained.  Ref: 14769373 Exp Neurol, 2003
- We are interested in the expression patterns of N methyl D aspartate  **NMDA**  and alpha amino 3 hydroxy 5 methyl 4 isoxazolepropionate  **AMPA**  receptors in DARPP 32 containing neurons, which may constitute morphological basis for interaction between dopamine and ionotropic glutamate receptors in dopaminoceptive cells.  Ref: 14751588 Brain Res, 2004
- Gly  **NMDA**  binding assays were performed to assess the selectivity of the reported compounds toward the  **AMPA**  receptor.  Ref: 14695840 J Med Chem, 2004
- **AMPA**  was more potent, increasing acetylcholine release at a lower concentration than  **NMDA** .  Ref: 14706791 Neuroscience, 2004

**Parallel relationship** (e.g. studied together, co-existance, homology, etc.)

- In anesthetized rats, the combined administration of the glutamatergic agonists N methyl D aspartic acid  **NMDA**  and alpha amino 3 hydroxy 5 methylisoxazole 4 propionic acid  **AMPA**  elicited ICP rises in the absence of DPN stimulation.  Ref: 14684562 Am J Physiol Regul Integr Comp PhysiolAm J Physiol Regul Integr Comp Physiol, 2004
- No effects on behavior and HPPT were obtained in  **NMDA**  6.25 ng, it or  **AMPA**  0.625 ng, it groups.  Ref: 14704116 Acta Pharmacol Sin, 2004
- To further illustrate the underlying mechanisms, calcium calmodulin dependent kinase IIalpha CaMKIIalpha inhibitor 1 N, O bis 5 isoquinolinesulfonyl N methyl L tyrosyl 4 phenylpiperazine KN62, CaM antagonist trifluoperazine TFP, N methyl D aspartate  **NMDA**  receptor antagonist dextromethorphan DEX,  **AMPA**  receptor antagonist dinitro quinoxaline 1H, 4H dione DNQX and L type voltage gated calcium channel L VGCC blocker nifedipine NIF, were respectively administrated to the rats 20 min prior to ischemia.  Ref: 14687884 Neurosci Res, 2004
- Effects of intrathecal  **NMDA**  and  **AMPA**  receptors agonists or antagonists on antinociception of propofol.  Ref: 14704116 Acta Pharmacol Sin, 2004
- These results indicate that glutamate can activate cholinergic basal forebrain neurons via both  **AMPA**  and  **NMDA**  ionotropic receptors but has a more modest effect on EEG activation.  Ref: 14706791 Neuroscience, 2004
- The combination of  **AMPA**  and  **NMDA**  did not produce any greater release than each drug alone, indicating that the effects of these two drugs on cholinergic neurons are not additive.  Ref: 14706791 Neuroscience, 2004
- OBJECTIVE To investigate the developmental profiles on surface expression and co localization of  **NMDA**  receptor clusters and  **AMPA**  receptor clusters on dendrite in cultured hippocampal neurons of rats.  Ref: 14712507 Zhejiang Da Xue Xue Bao Yi Xue Ban, 2003
- Defective spine formation in the mutants is associated with a drastic reduction in excitatory glutamatergic synapses and the clustering of  **NMDA**  and  **AMPA**  receptors.  Ref: 14691139 J Cell Biol, 2003
- Distribution of DARPP 32 positive neurons completely or partially overlapped with that of  **NMDA**  receptor or  **AMPA**  receptor immunoreactive ones in the frontal and parietal cortex, hippocampus and neostriatum, and neurons double labeled with DARPP 32 NR1, DARPP 32 NR2a b, DARPP 32 GluR1, DARPP 32 GluR2 3, or DARPP 32 GluR4 immunoreactivity were numerously observed.  Ref: 14751588 Brain Res, 2004
- During development, there is a switch from N methyl d aspartate  **NMDA**  to alpha amino 3 hydroxy 5 methyl 4 isoxazolepropionic acid  **AMPA**  mediated activity as the retinotectal map refines and visual function ensues.  Ref: 14769373 Exp Neurol, 2003
- These results support our hypothesis that glutamate, released upon stimulation of the genitals and acting at  **AMPA**  and  **NMDA**  receptors, is a potent activator of the spinal proerectile network.  Ref: 14684562 Am J Physiol Regul Integr Comp PhysiolAm J Physiol Regul Integr Comp Physiol, 2003
- In particular, the compounds were evaluated against audiogenic and maximal electroshock seizures MES test and pentetrazol PTZ seizures model, and all of them showed protective action.In addition, NBQX, benzodiazepines and THIQs... were also protective against clonic and tonic seizures and lethality induced by kainate,  **AMPA**  and ATPA, but were ineffective against  **NMDA**  induced seizures.  Ref: 14724045 Pharmacol Biochem Behav, 2004
- Double immunofluorescence was performed to visualize neurons showing coexpression of DARPP 32 with  **NMDA**  or  **AMPA**  receptor subunitsi.e., NR1, NR2a b, glutamate receptor subunit 1 GluR1, GluR2 3, and GluR4 in the forebrains of rats.  Ref: 14751588 Brain Res, 2004
- it is independent of the  **NMDA**  and  **AMPA**  receptor activity.  Ref: 14747522 Learn MemLearn Mem, 2004
- The study presents differential expression patterns of  **NMDA**  and  **AMPA**  receptors in DARPP 32 postive neurons in these forebrain regions.  Ref: 14751588 Brain Res, 2004
- Both agonists  **NMDA**  and  **AMPA**  reversed the antinociception of propofol.  Ref: 14704116 Acta Pharmacol Sin, 2004
- In the present experiments we administered an alpha amino 3 hydroxy 5 methyl 4 isoxazole propionic acid  **AMPA**  receptor antagonist CNQX, N methyl D aspartate  **NMDA**  receptor antagonist AP 5, or l type calcium channel blocker diltiazem directly into the ventral tegmental area VTA before each of four daily systemic cocaine injections in order to assess their influence on the initiation phase of behavioural sensitization.  Ref: 14725635 Eur J NeurosciEur J Neurosci, 2004
- 3H Kainate, 3H  **AMPA**  and  **NMDA**  displaceable 3H glutamate receptor binding were quantified in orbital cortex, cingulate cortex, medial frontal cortex, dorsolateral and dorsomedial striatum.  Ref: 14686906 Eur J Neurosci, 2003
- By applying informational measures to the simulation results of the dynamic behavior of  **AMPA** ,  **NMDA**  and GABA synaptic conductances we draw some conjectures about how the several neuronal synaptic elements are related to the initial stage of the digit induced reorganization of the hand map in the somatosensory cortex.  Ref: 14758065 J Comput NeurosciJ Comput Neurosci, 2003
- abolished by tetrodotoxin, but were unaffected by VIP receptor desensitization, hexamethonium,  **AMPA**  or  **NMDA**  glutamate receptor antagonists.  Ref: 14701723 Am J Physiol Gastrointest Liver PhysiolAm J Physiol Gastrointest Liver Physiol, 2003
- These data imply a possible change in the partnership of  **AMPA**  receptor subtype and  **NMDA**  receptor subtype at newly formed synapses during development.  Ref: 14712507 Zhejiang Da Xue Xue Bao Yi Xue Ban, 2003
- FLAG NR2B containing  **NMDA**  receptor clusters and GFP GluR2 containing  **AMPA**  receptor clusters expressed on membrane surface were then labeled in living neurons using anti FLAG mAb Cy3 conjugated anti mouse antibody and anti GFP pAb Alex488 conjugated anti rabbit antibody.  Ref: 14712507 Zhejiang Da Xue Xue Bao Yi Xue Ban, 2003
- Expression of NR1  **NMDA**  and GluR2  **AMPA**  receptor subtypes was examined immunohistochemically.  Ref: 14769373 Exp Neurol, 2003
- Activity dependent changes in expression and localization of the largest major isoform of the neural cell adhesion molecule NCAM180 and three subtypes of glutamate receptors predominantly expressed in the outer part of the molecular layer of the dentate gyrus of adult rats the  **NMDA**  receptor NR2A, the  **AMPA**  receptor GluR2 3, and the metabotropic glutamate receptor mGluR1 were investigated using postembedding immunogold labeling, and electron microscopy.  Ref: 14697660 Mol Cell Neurosci, 2003
- The effects of intrathecal  **NMDA** ,  **AMPA** , MK 801, NBQX, or NMDAR1 AS ODN on the antinociception of propofol were observed.  Ref: 14704116 Acta Pharmacol Sin, 2004
- Slow inward currents 273 35 pA peak amplitude, 381 25 ms latency, Vh = 73 mV representing evoked mGluR1 EPSCs were isolated in the presence of antagonists of  **AMPA** ,  **NMDA** , GABAA, GABAB, muscarinic and glycine receptors.  Ref: 14724196 J PhysiolJ Physiol, 2004
- AIM To study the effects of intrathecal it agonists and antagonists of N methyl D aspartate  **NMDA**  and alpha amino 3 hydroxy 5 methyl 4 isoxazole propionic acid  **AMPA**  receptors and NMDAR1 antisense oligodeoxynucleotides AS ODN on the antinociception of propofol.  Ref: 14704116 Acta Pharmacol Sin, 2004
- The selective ionotropic glutamate receptor agonists alpha amino dihydro 5 methyl 3 oxo 4 isoxazolepropionic acid  **AMPA**  and  **NMDA**  were ineffective in this respect.  Ref: 14770276 Naunyn Schmiedebergs Arch PharmacolNaunyn Schmiedebergs Arch Pharmacol, 2004
- Although  **AMPA**  kainate mediated activity persisted and GluR2 immunoreactivity increased transiently,  **NMDA**  receptor mediated activity was also consistently detected and NR1 expression increased.  Ref: 14769373 Exp Neurol, 2003
- Post mortem investigations have confirmed that glutamatergic  **NMDA** ,  **AMPA** , and kainate receptors are involved in the pathophysiology of schizophrenia.  Ref: 14722657 Nervenarzt, 2004
- The relevance of perirhinal cortical cholinergic and glutamatergic neurotransmission for taste recognition memory and learned taste aversion was assessed by microinfusions of muscarinic scopolamine,  **NMDA**  AP 5, and  **AMPA**  NBQX receptor antagonists.  Ref: 14747522 Learn MemLearn Mem, 2004
- In T8 spinalized rats, DPN stimulations were applied in the presence of D 2 amino 5 phosphonopentanoic acid D AP5, a competitive  **NMDA**  receptor antagonist, or of dioxo 6 nitro, tetrahydrobenzo f quinoxaline 7 sulphonamide NBQX, a  **AMPA**  kainate receptor antagonist, injected intrathecally at the lumbosacral level.  Ref: 14684562 Am J Physiol Regul Integr Comp PhysiolAm J Physiol Regul Integr Comp Physiol, 2004
- The highest concentrations of  **AMPA**  and  **NMDA**  tested produced small 25% but significant increases in high frequency activity.  Ref: 14706791 Neuroscience, 2004
- Some selected compounds were also tested for their functional antagonist activity at the  **AMPA**  and  **NMDA**  receptor ion channel complex.  Ref: 14695840 J Med Chem, 2004
- tetrahydrobenzo f quinoxaline 7 sulphonamide NBQX, an antagonist of alpha amino 3 hydroxy 5 methyl 4 isoxazolepropionate  **AMPA**  kainate receptors... an antagonist of N methyl D aspartate  **NMDA**  receptors, and verapamil, a blocker of voltage dependent calcium channels.  Ref: 14705143 J Neurosci Res, 2004
- Because  **AMPA**  and  **NMDA**  receptors, as well as l type calcium channels are calcium permeable, we also investigated the role of the calcium activated second messenger calcium calmodulin dependent protein kinase II CaM KII.  Ref: 14725635 Eur J NeurosciEur J Neurosci, 2004
- All the newly synthesized compounds were biologically evaluated for their binding at the Gly  **NMDA** ,  **AMPA** , and KA high affinity receptors.  Ref: 14695840 J Med Chem, 2004
- Although high rates 90 98% of DARPP 32 positive cells expressed  **NMDA**  receptors in all regions above, variant percentages of them expressing  **AMPA**  receptor subunits were observed among the cortex 54 90%, hippocampus 59 97% and neostriatum 14 97%.  Ref: 14751588 Brain Res, 2004
- These results suggest the presence of  **AMPA**  and  **NMDA**  receptors in association with motoneuron axons of larval zebrafish.  Ref: 14762151 J Neurophysiol, 2004
- During blockade of ionotropic glutamatergic  **AMPA**  and  **NMDA**  receptors, carbachol depolarized layer V neurons by 7.5 3.4 mV.  Ref: 14686907 Eur J Neurosci, 2003
- We suggest that the asynchronous activity reaching the optic tectum results in an inability to recapitulate the appropriate functional sequences of expression of  **NMDA**  and  **AMPA**  kainate receptors necessary to refine the retinotectal map.  Ref: 14769373 Exp Neurol, 2003
- AQ34 and AQ33b inhibited responses of NR1 NR2 receptors but did not inhibit responses of alpha amino 3 hydroxy 5 methyl 4 isoxazolepropionic acid  **AMPA**  receptors expressed from GluR1 or GluR2 Q, indicating that AQ34 and AQ33b are preferential  **NMDA**  antagonists.  Ref: 14764657 J Pharmacol Exp TherJ Pharmacol Exp Ther, 2003
- Differential expression of  **NMDA**  and  **AMPA**  receptor subunits in DARPP 32 containing neurons of the cerebral cortex, hippocampus and neostriatum of rats.  Ref: 14751588 Brain Res, 2004
- Stimulation of the prefrontal cortex afferent fibers to these VTA DA neurons in the presence of TTX, saclofen, and picrotoxin led to the conclusion that this input is mediated via  **NMDA**  and kainate  **AMPA**  receptors and may participate to induce behavioral sensitization to psychostimulants.  Ref: 14738903 Life Sci, 2004
- WIN reduced both  **NMDA**  and  **AMPA**  EPSCs, as well as miniature EPSCs mEPSCs, and increased the paired pulse ratio, indicating a presynaptic locus of its action.  Ref: 14715937 J Neurosci, 2004
- Receptor function was examined using  **NMDA**  and  **AMPA**  kainate antagonists during in vitro tectal recording of visually evoked post synaptic extracellular responses.  Ref: 14769373 Exp Neurol, 2003
- 100 or 1000 microM or a combination of  **AMPA**  10 microM and  **NMDA**  100 microM was administered to the basal forebrain using reverse microdialysis.  Ref: 14706791 Neuroscience, 2004
- The present findings also suggest that increased calcium influx through  **AMPA**  receptors,  **NMDA**  receptors and l type calcium channels on dopaminergic neurons in the VTA contributes significantly to the initiation of behavioural sensitization by amplifying calcium signalling through CaM KII.  Ref: 14725635 Eur J NeurosciEur J Neurosci, 2004
- Surface expression and co localization of  **NMDA**  receptor and  **AMPA**  receptor on dendritic tree of hippocampal neurons in culture .  Ref: 14712507 Zhejiang Da Xue Xue Bao Yi Xue Ban, 2003
